# Supplementary material for: The mosaic of Rickettisae from the Caatinga Biome, Brazil: New records and first interactions
Source: PLoS Negl Trop Dis. 2025 Nov 10;19(11):e0013670. doi: 10.1371/journal.pntd.0013670 (PMC12614789; doi:10.1371/journal.pntd.0013670)
Supplement: Attachment — Submitted filename: PNTD-D-25-00108.pdf [file pntd.0013670.s001.pdf]

PLOS Neglected Tropical Diseases

The Mosaic of Rickettisae in Caatinga biome, Brazil: New interactions between Rickettsia parkeri and Amblyomma Parvum

--Manuscript Draft--

**Manuscript Number:**	PNTD-D-25-00108
**Full Title:**	The Mosaic of Rickettisae in Caatinga biome, Brazil: New interactions between Rickettsia parkeri and Amblyomma Parvum
**Short Title:**	Expansion of Rickettsiae in northeastern Brazil
**Article Type:**	Research Article
**Keywords:**	Rickettsiae; biodiversity; Rickettsia parkeri; Rickettsia amblyommatis; Spotted Fever, Rickettsia parkeri; Amblyomma parvum; Rhipicephalus linnaei; Northeastern Brasil; Ceara, Caatinga biome; Parasitology
**Abstract:**	Rickettsiosis are infections caused by bacteria of the genus Rickettsia, transmitted by hematophagous ectoparasites, mainly ticks and fleas. Due to the nature of their vectors, Rickettsiae have wordlwide distribution, occurring in all regions of Brazilian territory and being distributed across its various biomes. Among the most relevant rickettsiosis in Brazil is Spotted Fever (SF), a potentially lethal zoonosis that requires mandatory notification to the Ministry of Health. Although cases of SF have been confirmed in Brazil, the majority were concentrated in the Southeast region, with the epidemiological situation in the Northeast being poorly understood. In 2010, the first cases were confirmed in the state of Ceará; however, there is still a lack of knowledge regarding the true presence and identity of circulating Rickettsia species in the state. The aim of this study was to analyze the circulation and specific identification of Rickettsia species in the state of Ceará, as well as to identify potential vector species, thereby contributing to the development of an eco-epidemiological profile of the state and enhancing public health support. For the research, genomic DNA samples extracted from ticks originating from places with suspected cases were used; these ticks were previously taxonomically identified. These samples span from the years 2017 to 2023 and are available from the sample bank of Laboratório de Carrapatos e Outros Artrópodes Ápteros (LAC-FIOCRUZ). Samples were analyzed using PCR to detect infection by Rickettsia spp. (characterizing genus and specific group), with positive samples undergoing further analysis using new markers for specific characterization and genomic sequencing. The results detected for the first time the presence of Rickettsia amblyommatis and R. asembonensis in the state, as well as indicating a geographical expansion of other species of the genus, with records in new regions. Additionally, the first interaction between Rickettsia parkeri and Amblyomma parvum was registered.
**Additional Information:**	
**Question**	**Response**
**Government Employee**<br><br>Are you or any of the contributing authors an employee of the United States government?<br><br>Manuscripts authored by one or more US Government employees are not copyrighted, but are licensed under a CC0 Public Domain Dedication, which allows unlimited distribution and reuse of the article for any lawful purpose. This is a	No - No authors are employees of the U.S. government.

Powered by Editorial Manager® and ProduXion Manager® from Aries Systems Corporation

legal requirement for US Government employees.<br><br>This will be typeset if the manuscript is accepted for publication.	
**Financial Disclosure**<br><br>Enter a financial disclosure statement that describes the sources of funding for the work included in this submission. Review the submission guidelines for detailed requirements. View published research articles from *PLOS Neglected Tropical Diseases* for specific examples.<br><br>This statement is required for submission and **will appear in the published article** if the submission is accepted. Please make sure it is accurate.<br><br>**Funded studies**<br>Enter a statement with the following details:<br>• Initials of the authors who received each award<br>• Grant numbers awarded to each author<br>• The full name of each funder<br>• URL of each funder website<br>• Did the sponsors or funders play any role in the study design, data collection and analysis, decision to publish, or preparation of the manuscript?<br><br>Did you receive funding for this work?	The author(s) received no specific funding for this work.
**Competing Interests**<br><br>On behalf of all authors, disclose any competing interests that could be perceived to bias this work.<br><br>This statement will be typeset if the manuscript is accepted for publication.<br><br>Review the instructions link below and PLOS NTDs' competing interests policy to	The authors have declared that no competing interests exist.

Powered by Editorial Manager® and ProduXion Manager® from Aries Systems Corporation

determine what information must be disclosed at submission.	
**Data Availability**<br><br>Provide a **Data Availability Statement** in the box below. This statement should detail where the data used in this submission can be accessed. This statement will be typeset if the manuscript is accepted for publication.<br><br>Before publication, authors are required to make all data underlying their findings fully available, without restriction. Review our PLOS Data Policy page for detailed information on this policy. Instructions for writing your Data Availability statement can be accessed via the Instructions link below.	The data supporting these finds will be in the process of deposition on GenBank within the week. Any further doubts and inquiries, my e-mail is available (joaofgazeta@hotmail.com)

Powered by Editorial Manager® and ProduXion Manager® from Aries Systems Corporation

 Click here to access/download;Manuscript;Expansion of Rickettsiae in Northeasten Brazil.docx

The Mosaic of Rickettisae in Caatinga biome, Brazil: New interactions between *Rickettsia parkeri* and *Amblyomma Parvum*

João F. Audi Gazeta[1], Ana Beatris Pais Borsoi[2], Karla Bitencourth[2], Mariana Guimarães Côrtes[2], Ingrid Benevides Machado[2], Robson Cavalcante[3], Gilberto Salles Gazeta[2], Nathalie Costa da Cunha[2]

[1]Departamento de Saúde Coletiva Veterinária e Saúde Pública, Universidade Federal Fluminense, Niterói, Rio de Janeiro, Brazil

[2] Laboratório de Referência Nacional em Vetores das Riquetsioses, Instituto Oswaldo Cruz (IOC-FIOCRUZ), Rio de Janeiro, Rio de Janeiro, Brazil

[3]Laboratório de Vetores, Reservatórios e Animais Peçonhentos Dr. Thomaz Corrêa Aragão (LAVRAP), Fortaleza, Ceará, Brazil.

*Corresponding author

E-mail: joaofgazeta@hotmail.com (JFA)

gsgazeta@ioc.fiocruz.br (GSG)

Abstract

Rickettsiosis are infections caused by bacteria of the genus *Rickettsia,* transmitted by hematophagous ectoparasites, mainly ticks and fleas. Due to the nature of their vectors, Rickettsiae have wordlwide distribution, occurring in all regions of Brazilian territory and being distributed across its various biomes. Among the most relevant rickettsiosis in Brazil is Spotted Fever (SF), a potentially lethal zoonosis that requires mandatory notification to the Ministry of Health. Although cases of SF have been confirmed in Brazil, the majority were concentrated in the Southeast region, with the epidemiological situation in the Northeast being poorly understood. In 2010, the first cases were confirmed in the state of Ceará; however, there is still a lack of knowledge regarding the true presence and identity of circulating *Rickettsia* species in the state. The aim of this study was to analyze the circulation and specific identification of Rickettsia species in the state of Ceará, as well as to identify potential vector species, thereby contributing to the development of an eco-epidemiological profile of the state and enhancing public health support. For the research, genomic DNA samples extracted from ticks originating from places with suspected cases were used; these ticks were previously taxonomically identified. These samples span from the years 2017 to 2023 and are available from the sample bank of Laboratório de Carrapatos e Outros Artrópodes Ápteros (LAC-FIOCRUZ). Samples were analyzed using PCR to detect infection by *Rickettsia* spp. (characterizing genus and specific group), with positive samples undergoing further analysis using new markers for specific characterization and genomic sequencing. The results detected for the first time the presence of *Rickettsia amblyommatis* and *R. asembonensis* in the state, as well as indicating a geographical expansion of other species of the genus Rickettsiae into Northeastern Brazil, with records in new regions. Additionally, the first interaction between *Rickettsia parkeri* and *Amblyomma parvum* was registered, possibly changing our understanding of interactions between R. pa[illegible]i and A. parv[illegible]

Author Summary

Rickettsiae are bacteria spread mainly by ticks and fleas that can cause diseases in humans and animals. This study focused on identifying different species of Rickettsia and their potential tick carriers in the state of Ceará, Brazil. We examined 1,359 samples from ticks collected on animals and in the environment. After initial testing, 82 samples showed signs of Rickettsia bacteria, and we successfully identified specific species in 37 of them using genetic analysis.

Notably, this research found *Rickettsia amblyommatis* and *Rickettsia asembonensis* circulating in the region for the first time, adding to our understanding of local bacteria and their tick hosts. We also discovered a new association between the tick *Amblyomma parvum* and *Rickettsia parkeri*, a species linked to mild human infections.

These findings help map which ticks carry which bacteria, offering insights into how Rickettsiae spread in the environment. This is crucial for public health planning, as some species can cause illness in humans. By identifying these bacteria and their carriers, this research contributes to a better understanding of disease risks in Ceará and similar regions worldwide.

Introduction

Rickettsioses are zoonotic infectious diseases caused by bacteria of the genus *Rickettsia*, pleomorphic organisms with an obligate intracellular life cycle, transmitted by wingless arthropods with global distribution [1].Among the known forms of rickettsioses, the Spotted Fever Group (SFG) zoonoses, primarily represented in Brazil by the species *Rickettsia rickettsii* and *Rickettsia parkeri*, hold the greatest significance and distribution. These diseases are considered of mandatory notification in Brazil due to their acute nature and potentially lethal outcomes [2].

Species of the genus *Rickettsia* are currently classified within four groups: Ancestral Group, Transitional Group, Typhus Group, and Spotted Fever Group (SFG). Except for the Ancestral Group, all others include pathogenic species capable of causing diverse clinical conditions in humans and animals. Notably, *Rickettsia prowazekii* and *R. typhi* are the causative agents of epidemic typhus and murine typhus, respectively, while *Rickettsia rickettsii* and *R. parkeri* are the primary species linked to spotted fever in the Am[illegible]as. *Rickettsia* species from the Ancestral and Spotted Fever Groups are predominantly associated with ticks, whereas those from the Typhus and Transitional Groups are more commonly linked to mites, fleas, and lice [3,4].

In Brazil, the presence of vectors capable of maintaining and transmitting rickettsioses has been confirmed in all regions, along with the detection of *Rickettsia* spp., leading to the implementation of the **National Environmental Surveillance System for Rickettsioses and Other Tick-Borne Diseases**. This initiative has enhanced the understanding of the country's epidemiological situation regarding this public health concern. The primary vectors of rickettsioses in Brazil are ticks, blood-feeding ectoparasites represented by various genera and species, such as *Amblyomma spp.*, *Rhipicephalus spp.*, and *Dermacentor spp.*, all of which have been associated with

Rickettsia spp. Other potential vectors of *Rickettsia* spp. in Brazil include fleas, lice, and mites [5]

Spotted Fever in Brazil: Overview

With the establishment of Spotted Fever as a notifiable disease and the implementation of the Surveillance Network, new studies on its true distribution and epidemiological status began to emerge. Initially, these studies were more focused on the South and Southeast regions of Brazil but eventually expanded to previously neglected areas, particularly the Midwest, North, and Northeast regions. In light of this increased surveillance, data gathered from the Notifiable Diseases Information System (SINAN/Ministry of Health) reveals that the Southeast region has experienced the highest number of confirmed cases in the historical series (2007–2024), totaling 1,924 cases (approximately 113 cases per year). The South region follows with 726 confirmed cases (approximately 43 cases per year), while the Northeast reports 56 cases, the Midwest has 38, and the North, the least affected, recorded 10 cases during this period [6].

In this context, the most severe form of spotted fever, associated with *Rickettsia ricketssii* infection, is recorded in the Southeast region and the northern part of Paraná, a state in the South region of the country [7] In these areas, predominantly composed of Cerrado (Tropical Savanna) and anthropized Atlantic Rainforest, the transmission of *R. rickettsii* is mainly attributed to the tick *Amblyomma sculptum*, especially in environments with the presence of equids and capybaras, the main hosts of this vector [8]. Additionally, a study conducted by Pacheco et al. [9] in the state of Minas Gerais identified *Rhipicephalus linnaei* ticks (previously classified as *R. sanguineus*) naturally infected with *R. rickettsii* and capable of transmission, with the etiological agent being isolated and cultured in the laboratory.

In contrast, a focus of Spotted Fever was identified in an area with lower anthropogenic impact in the metropolitan region of São Paulo. In this scenario, the tick *Amblyomma aureolatum* stands out as the primary vector of *R. rickettsii*, while dogs appear to play a significant role in the epidemic cycle of the disease. This distinct dynamic reflects the ecological and behavioral differences that can influence the transmission of Spotted Fever in various regions of the country [8,10].

Although the epidemiological landscape in certain regions of Brazil is relatively well characterized, the understanding of Spotted Fever (SF) in other environments remains incipient. For instance, in the Midwest region of Brazil, there have been reports of cases with notable clinical differences, but without confirmation of the vector species and rickettsiae involved. Likewise, in other areas of the country identified as new hotspots for SF, such as Maranhão, Rondônia, Pernambuco, Alagoas, Paraíba, and Sergipe, further studies are needed to clarify the epidemiological profile of the region's rickettsiae [7,11].

In the Northeast region, in which stands the Caatinga biome, the eco-epidemiological landscape of spotted fever, as well as of rickettsioses in general, tough somewhat more familiar, remains relatively unclear. In 2010, the Ceará State Health Secretariat reported the first confirmed cases of spotted fever in the Baturité Massif and the capital city, Fortaleza— both of which are tropical climates and fall within the Atlantic Forest biome, in contrast to the majority of the state, which is characterized by a semi-arid climate and the Caatinga biome. In 2016, further studies confirmed the circulation of *Rickettsia spp*. from different groups in the state, as well as the presence of potential vectors [12]. Also, recent findings from Cavalcante et al. [13] now indicate the presence of rickettsiae in several municipalities within the Ceará semi-arid region. Therefore, this study aimed to analyze the epidemiological situation of rickettsioses in the state, with a particular focus on the biodiversity of the etiological agent involved and its main vectors.

METHODS

The work was carried out at the Molecular Epidemiology Laboratory, at the Veterinary School of the Federal Fluminense University (Laboratório de Epidemiologia Molecular, Faculdade de Veterinária - Universidade Federal Fluminense); the Tick and Other Acarine Arthropods Laboratory (Laboratório de Carrapatos e Outros Artrópodes Ápteros, LAC – Fiocruz); and the Fiocruz Technological Platforms for Genomic Sequencing Network (PDTIS).

Collection and Screening of Ectoparasite Samples

All samples used in this study were obtained from the LAC sample repository, through the National Environmental Surveillance System for Rickettsioses and Other Tick-Borne Diseases (RNVADTC). The capture of potential vector samples was carried out during environmental surveillance and case investigations for Spotted Fever between 2017 and 2023 by the Health Secretariat of the State of Ceará (SESA/CE). Potential vectors were gathered from vertebrate hosts and the environment using techniques such as flannel dragging and direct picking.

Captured ticks were then sent to the Central Laboratory of the State of Ceará (LACEN/CE) and subsequently forwarded to the LAC, following the protocol established by the RNVADTC. Taxonomic identification was performed under a stereomicroscope using specific identification keys: Amorim and Serra-Freire [14] for the larval stage, Martins et al. [15] for the nymphal stage, and Barros-Battesti et al. [16] and Dantas-Torres et al. [17] for the adult stage.

Detection and Characterization of Bioagents

The properly identified specimens were aliquoted into 1.5 mL microtubes, either individually (for nymphs and adults) or grouped (for larvae). Subsequently, the samples underwent genomic DNA (gDNA) extraction following the enzymatic extraction protocol previously described by Aljanabi and Martinez [18] in 1997.

The DNA was then subjected to amplification through screening PCR (Polymerase Chain Reaction) using genus-specific and group-specific markers for SFG Rickettsiae: *gltA* (CS2-78/CS2-323) and *ompA* (Rr.190.70p/Rr190.602), respectively [19]. Samples that amplified at least one of these genes were further analyzed using specific characterization markers for Rickettsiae from the spotted fever and typhus groups: *htrA* (17Kd1/17Kd2) and *ompB* (SFG-IF/SFG-TG IR) [20, 21].

The shift in primers used was due to their characteristics, as a significant portion of the samples had been stored for years, resulting in the natural degradation of genetic material. Consequently, older samples no longer amplified with the original primers. Thus, new PCRs for both and new positive samples were conducted using Nested primers, chosen for their higher detection sensitivity, ensuring uniform results across all samples.

After the amplification of each gene, the samples were subjected to electrophoresis on a 2% agarose gel, stained with ethidium bromide [22]. The gel visualization and photographic documentation were performed using the MiniBis Pro system (DNR Bio® - Imaging Systems Ltd). Samples that amplified the expected fragment were purified using the Wizard® SV Gel and PCR Clean-up System (Promega®) kit, following the manufacturer's recommendations. Subsequently, the purified amplicons were sent for sequencing of the obtained genetic material.

DNA sequencing was performed on the DNA sequencing platform (PDTIS) at FIOCRUZ/RJ. Sequencing reactions were carried out using the BigDye™ Terminator v3.1 Cycle Sequencing Kit (Applied Biosystems, Carlsbad, California, USA), following the manufacturer's recommendations. The same primers used in the PCR were employed in these reactions for sequence determination in both directions (3'-5' and 5'-3'). Subsequently, the samples were precipitated, resuspended in formamide, and applied to an ABI 3730xl automatic sequencer (Applied Biosystems, Carlsbad, California, USA) for sequence reading.

All obtained sequences for each gene were manually edited, and consensus sequences were generated using the ChromasPro 1.5 program (Technelysium Pty Ltd, Tewantin, Qld, Australia). Initially, the DNA sequences were identified by similarity assessment, through a comparative analysis with sequences deposited in GenBank, using BLASTN (Basic Local Alignment Search Tool - Nucleotide). After editing, the sequences obtained for each gene were automatically aligned using the ClustalW multiple alignment algorithm [23], available in the MEGA 6.0 program [24]. All alignments were manually inspected, and the protein-coding genes were translated into amino acids to check for the presence of pseudogenes and confirm homology, with no stop codons being observed.

To improve upon the understanding of the identification of rickettsiae detected in the Ixodidae ticks studied in Ceará, a concatenated phylogenetic reconstruction of fragments from the ompB (411 bp) and htrA (434 bp) genes was performed. The SeaView program [25] was used to concatenate the alignments of the investigated genes. Phylogenetic analysis, using maximum likelihood analysis, was carried out in the PhyML 3.0 program [26] with the evolutionary model T92+G (through the Bayesian Information Criterion) indicated by MEGA 6.0, which best fitted the analyzed dataset. Statistical

support values for internal branches were estimated with the aLRT test (approximate likelihood ratio test) using 1000 replicates [27].

RESULTS

During the study period (2017–2023), environmental surveillance activities and investigations of suspected Spotted Fever cases were conducted by the Health Secretariat of the State of Ceará (SESA/CE), as well as municipal and regional health secretariats across 14 municipalities in the state. The distribution of infested hosts recorded during these activities is presented in **Table 1** below.

Table 1 - Absolute (n) and relative (%) frequency of the main vertebrate hosts found with potential vectors for *Rickettsia* sp. during previous surveillance activities in different municipalities of the state of Ceará, from 2017 to 2023.

Vertebrate Host	n	%
Canis familiaris	338	75,11%
Bos taurus	63	14%
Homo sapiens	19	4,22%
Felis catus	9	2%
Equus caballus	9	2%
Gallus galuus	6	1,33%
Cerdocyon thous	3	0,67%
Coendou sp.	3	0,67%
**Total**	**450**	**100%**

A total of 3,098 ticks with vector potential were collected during the study period, comprising 1,562 females (50.4%), 1,125 males (36.3%), 378 nymphs (12.2%), and 33 larvae (1%). Of this total, 1,35[illegible] ([illegible]44%) were processed according to the protocol established by LAC - FIOCRUZ, of which 82 (6%) amplified for *Rickettsia* sp. fragments during preliminary screening for gltA and/or ompA. The distribution of species in relation to hosts, along with their respective absolute and relative frequencies, can be found in Table 2 (Ixodidae/Argasidae) below

Table 2 - Absolute (n) and relative (%) frequency of ticks (Ixodidae/Argasidae) captured during previous surveillance activities in different municipalities of the state of Ceará, from 2017 to 2023.

Host	Ixodidae								Argasidae		
	A. longirostre	*A. nodosum*	*A. ovale*	*A. parvum*	*A. rotundatum*	*D. nitens*	*R. linnaei*	*R. microplus*	*Argas miniatus*	*O. rietcorreai*	*O. talaje*
	n (%)	n (%)	n (%)	n (%)	n (%)	n (%)	n (%)	n (%)	n (%)	n (%)	n (%)
Environment	6 (18,75%)	-	1 (3,22%)	15 (39,5%)	2 (50%)	-	185 (18,3%)	-	25 (80%)	8 (100%)	-
Canis familiaris	-	2 (100%)	26 (83,88%)	13 (34,20%)	-	-	816 (80,7%)	6 (3,05%)	-	-	-
Bos taurus	-	-	-	2 (5,26%)	-	-	4 (0,40%)	189 (95,94%)	-	-	-
Homo sapiens	-	-	3 (9,68%)	5 (13,15%)	2 (50%)	-	-	2 (1,01%)	-	-	3(100%)
Felis catus	-	-	-	1 (2,63%)	-	-	4 (0,40%)	-	-	-	-
E. caballus	-	-	-	-	-	5 (100%)	-	-	-	-	-
Gallus galuus	-	-	-	-	-	-	-	-	6 (20%)	-	-
C. thous	-	-	1 (3,22%)	2 (5,26%)	-	-	2 (0,20%)	-	-	-	-
Coendou sp.	26 (81,25%)	-	-	-	-	-	-	-	-	-	-
Total	32 (100%)	2 (100%)	31 (100%)	38 (100%)	4 (100%)	5 (100%)	1011 (100%)	197 (100%)	31(100%)	8(100%)	3 (100%)

[illegible]Amblyomma; B: *Boophilus;* R: *Rhipicephalus;* D: *Dermacentor*; Ca: Canis; Ce: Cerdocyon; E: Equus; O: *Ornithodoros*;

Among the 1,359 samples processed during the study period, 82 (6%) tested positive during screening, showing amplification for the target genes *gltA* and/or *ompA*. From the 82 samples that amplified during screening, genetic sequencing and phylogenetic analysis were successfully performed on 37, representing 2.72% of the total and 45.12% of those that amplified the expected fragment. Of these 37 sequenced samples, 24 were analyzed for the ompB gene, from a total of 25 amplified by Nested PCR, and 21 were analyzed for the htrA gene, from a total of 23 amplified by Nested PCR.

Following sequencing and phylogenetic analysis, a concatenated phylogenetic tree was constructed using the aforementioned fragments (Figure 1 below). This analysis revealed the presence of five circulating *Rickettsia* species in the studied regions: *Candidatus Rickettsia andeanae* (n=16), *Rickettsia amblyommatis* (n=9), *Rickettsia parkeri* strain Atlantic Rainforest (n=7), *Rickettsia felis* (n=4), and *Rickettsia asembonensis* (n=1).

Fig. 1 - Concatenated phylogeny, inferred through maximum likelihood analysis with the T92+G evolutionary model, of partial DNA sequences from the ompB + htrA genes of rickettsiae (411bp + 434bp) detected in ticks from Ceará, Brazil (highlighted with a star), in comparison to Rickettsia sequences retrieved from GenBank. The numbers on the branches represent statistical support values (70% cut-off). Stars indicate sequences obtained in the present study.

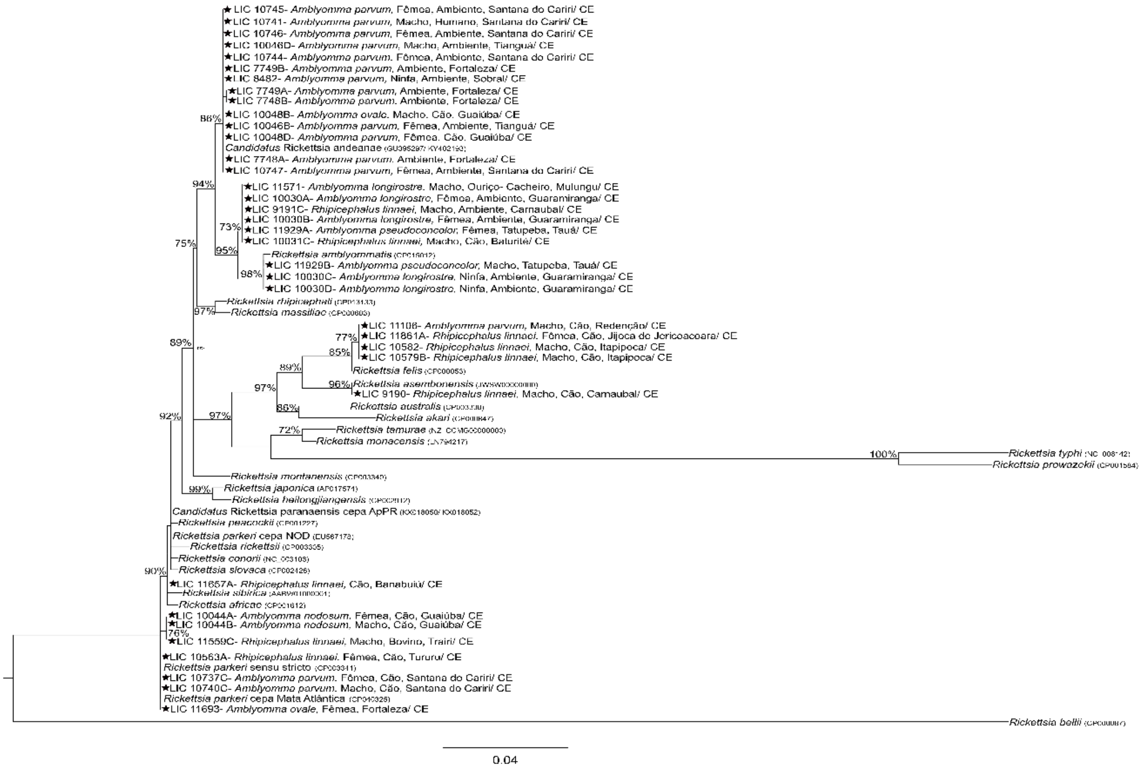

The *Rickettsia* species were detected in a variety of vectors (six species) originating from different hosts, as shown in Table 3 and 4 (which include municipalites associated with Rickettisae) below. Notably, of the 37 sequences obtained, 14 (47.82%) were found in domestic dogs, 7 (29.17%) were from vectors collected directly from the environment and not associated with specific hosts, 2 (8.69%) were obtained from armadillos (*Euphractus sexcinctus*), 2 (8.69%) from cattle (*Bos taurus*), 1 (4.34%) from a hedgehog (*Coendou prehensilis)*, and 1 (4.34%) from a specimen of *Amblyomma parvum*, which was found parasitizing a human.

Tab. 3- Distribution of sequenced samples correlating vectors, hosts, and species of *Rickettsia spp*. identified during the period from 2017 to 2023

Vetores	Hospedeiros	*Rickettsia spp.*
Rhipicephalus linnaei	*Canis familiaris*<br>*Bos taurus*	*Rickettsia parkeri*<br>*Rickettsia amblyommatis*<br>*Rickettsia felis*<br>*Rickettsia asembonensis*
Amblyomma parvum	*Canis familiaris*<br>*Homo sapiens*	*Rickettsia parkeri*<br>*Rickettsia felis*<br>*Ca. R. Andeanae*
Amblyomma longirostre	*Coendou prehensilis*	*Rickettsia amblyommatis*
Amblyomma nodosum	*Canis familiaris*	*Rickettsia parkeri*
Amblyomma ovale	*Canis familiaris*	*Rickettsia parkeri*
Amblyomma pseudoconcolor	*Euphractus sexcinctus*	*Rickettsia amblyommatis*

Tab. 4- Distribution of *Rickettsia* Species, Associated Vectors, Hosts, and Localities in Ceará, Brazil (2017–2023)

Rickettsiae	Vectors	Hosts	Municipalities (n)
Candidatus Rickettsia andeanae	*A. parvum*	Environment	Santana do Cariri (n=4)<br>Fortaleza (n=4)<br>Tianguá (n=2)<br>Sobral (n=1)
		Canis familiaris	Guaiuba (n=1)
		Homo Sapiens	Santana do Cariri (n=1)
	Rh. linnaei	Environment	Carnaubal (n=1)
		Canis familiaris	Baturité (n=1)
	A. ovale	*Canis familiaris*	Guaiuba (n=1)
Rickettsia amblyommatis	*A. longirostre*	Environment	Guaramiranga (n=4)
		Coendou prehensilis	Mulungu (n=1)
	A. pseudoconcolor	*Euphractus sexcinctus*	Tauá (n=2)
	Rh. linnaei	*Canis familiaris*	Baturité (n=1)
Rickettsia parkeri	*A. nodosum*	*Canis familiaris*	Guaiuba (n=2)
	A. parvum	*Canis familiaris*	Santana do Cariri (n=2)
	A. ovale	-	Fortaleza (n=1)
	Rh. linnaei	*Canis familiaris*	Trairi (n=1)
		Bos taurus	Tururu (n=1)
Rickettsia felis	*A. parvum*	*Canis familiaris*	Itapipoca (n=1)
	Rh. linnaei	*Canis familiaris*	Redenção (n=2)<br>Jijoca de Jericoacoara (n=1)
Rickettsia asembonensis	*Rh. linnaei*	*Canis familiaris*	Carnaubal (n=1)

A.: Amblyomma

The collected samples were distributed from 14 municipalities across various bi[illegible]s, microregions, and mesoregions of the state. The study revealed the presence of rickettsiae in three new municipalities: Tauá, Trairi, and Jijoca do Jericoacoara. Additionally, in the regions of Guaiuba, Santana do Cariri, Itapipoca, Tururu, and Tianguá, the research contributed to expanding the knowledge of circulating rickettsiae by identifying specific species in areas where rickettsiae were previously detected but not identified on a species level. In the remaining municipalities, the research contributed to expanding knowledge on the circulating rickettsiae, revealing the presence of new species in areas where rickettsiae had already been detected previously.

DISCUSSION

Among the five *Rickettsia* species identified in this study, *R. felis* and *R. asembonensis* are classified within the Transitional Group, while *Rickettsia parkeri*, *Rickettsia amblyommatis*, and *Candidatus Rickettsia andeanae* are members of the Spotted Fever Group (SFG). The detection of *R. parkeri*, *R. felis*, and *Ca. R. andeanae* corroborates previous findings reported by Moerbeck et al. [12,28] and Cavalcante et al. [13]. Notably, there have been no prior records of *R. asembonensis* or *R. amblyommatis* in studies conducted within the state, thereby establishing this study as the first to document the presence of these organisms in the region.

Spotted Fever Group Rickettsiae

Rickettsia parkeri

Rickettsia parkeri was identified in three distinct genetic clusters: *Rickettsia parkeri sensu stricto* (s.s.), *R. parkeri* Mata Atlântica strain, and *R. parkeri* NOD strain. These clusters represent clades within the species, of which *R. parkeri s.s.* and *R. parkeri* Atlantic

Rainfores strain have confirmed pathogenicity, causing a mild form of spotted fever. In contrast, the pathogenicity of the NOD strain remains unknown [29,30].

It is important to note that during the phylogenetic analysis, it was observed that there is no available sequence in GenBank for *Rickettsia parkeri* strain NOD for the analyzed *ompB* gene fragment, which limited the analysis of the results, since samples LIC 10044B, LIC 10044A, and LIC 11559C only successfully sequenced the *ompB* fragment. Consequently, while it was possible to use the available data for the conserved *htrA* gene [31], the results may not fully reflect the detection of *Rickettsia parkeri* strain NOD in samples that only amplified the *ompB* fragment. Thus, considering the epidemiological knowledge of Spotted Fever in the studied region and the results from phylogenetic analysis, a short but perceptible genetic distance was observed between samples LIC 10044B, LIC 10044A, and LIC 11559C and the *R. parkeri* Mata Atlântica strain samples. This proximity raises the hypothesis that these samples may be infected with *R. parkeri* strain NOD, rather than the Mata Atlântica strain indicated by the phylogenetic tree.

Upon analyzing the results, it became evident that the tick species *Rhipicephalus linnaei* was the most frequently collected during the study (Tab. 2), with a total of 674 samples processed. From these, 45 (6.67%) successfully amplified during the screening phase conducted throughout the study period. However, due to the exhaustion of certain materials and the degradation of stored DNA, only 10 samples (1.48% of the total) were successfully sequenced. Notably, this species exhibited the greatest diversity of associated *Rickettsia* spp., including *Rickettsia parkeri* (Atlantic Rainforest strain), which, alongside *R. rickettsii*, is recognized as one of the primary causative agents of Spotted Fever in Brazil [2].

Although the finding of *R. parkeri* in *Rhipicephalus linnaei* does not necessarily serve as conclusive evidence of its potential as a vector for Spotted Fever, previous studies

conducted in Brazil have demonstrated the ability of *Rickettsia* from the Spotted Fever Group (e.g., *Rickettsia rickettsii*) to be transmitted to vertebrate mammals through *Rh. linnaei* bites, as well as through transstadial and transovarial transmission. These studies have classified *Rhipicephalus linnaei* as a competent vector for Spotted Fever, at least in laboratory settings [9,32,33].

The findings of Piranda et al. [32] in a laboratory experiment demonstrated the ability of dogs to act as amplifiers of rickettsiae. However, it was shown that less than 40% of the ticks used in the experiment became infected when feeding on dogs infected with *Rickettsia rickettsii*. In parallel, the study by Pacheco et al. [9] found a 100% infection rate in the offspring of naturally infected female *Rh. linnaei* collected directly from free-roaming dogs, demonstrating the natural maintenance capability of the rickettsiae's GFM enzootic cycle. Similarly, studies conducted in Mexico support these findings, with *Rh. sanguineus* s.l. (i.e., *Rh. linnaei*) considered the primary species associated with the transmission of Spotted Fever by *R. rickettsii* in the northern part of the country, with dogs as the main host [33,34].

Simultaneously, we also observed the presence of *Rickettsia parkeri* in *Amblyomma nodosum* and *Amblyomma ovale*, in agreement with the findings of Moerbeck et al. [12,28] in the state of Ceará. However, the presence of this agent in *Amblyomma parvum* had not yet been documented in the country, making this the first report of an interaction between these species. This interaction is particularly noteworthy due to the low parasitic specificity and markedly anthropophilic behavior exhibited by this ixodid, as highlighted by Guglielmone and Robbins [35]. Nonetheless, caution is warranted when discussing this topic, given that the species is not recognized as a vector for rickettsiae. Furthermore, it is also relevant that the infected *A. parvum* specimens were collected from hosts in the

municipality of Santana do Cariri, where, to date, no species-specific confirmation of any *Rickettsia* has been reported [13].

These data, coupled with the relatively low number of *Amblyomma ovale* ticks collected and processed during the study (Tab. 2), the low prevalence of *Rickettsia parkeri* among these specimens—only one tick tested positive for the agent (3.22% of the total processed)—and considering that the positive specimen was identified in a municipality where *R. parkeri* presence had already been previously confirmed (Fortaleza), suggest not only a potential role for *A. parvum* in the *R. parkeri* cycle but also a likely significant involvement of *Rhipicephalus linnaei* in the maintenance of the enzootic and epidemiological cycle of *R. parkeri*, at least in the state of Ceará. However, further studies are necessary to clarify and confirm this hypothesis.

Candidatus Rickettsia andeanae

First reported in Brazil in 2014 by Nieri-Bastos et al., *Ca. Rickettsia andeanae* is a bacterium from the spotted fever group (SFG) with a wide geographical distribution, found infecting different tick species in several countries such as Peru, Paraguay, Argentina, Chile, the United States, and Brazil [36,37,38]. In Brazil, this rickettsia is commonly associated with *Amblyomma parvum* ticks, being recorded in at least four Brazilian biomes: Cerrado, Pantanal, Atlantic Forest, and Caatinga [12,39,40], a finding corroborated by the present study, in which 13 (34.21%) of the 38 specimens of *A. parvum* collected were sequenced for the agent in question. Furthermore, its presence was also detected in two *Rhipicephalus linnaei* ticks and one *Amblyomma ovale* specimen, consistent with the findings of Krawczack et al. [38].

Although considered to have undetermined pathogenicity, Krawczak et al. [38] suggests that *Ca. R. andeanae* may not be pathogenic at all, citing the studies of Ferrari

et al. [41], which demonstrated the bacterium's apparent inability to multiply in mammalian cells in laboratory experiments with Vero cells, and Grasperge et al. [42], which observed its apparent lack of transmission and reproduction in the skin of mice. Nonetheless, *Ca. R. andeanae* presents particularities that warrant further investigation, such as its association with various tick species and a potential negative correlation with *Rickettsia parkeri*, as suggested by Paddock et al. [43] and Krawczak et al. [38].

This correlation gains additional significance when considering that ixodid ticks are known to be unable to simultaneously transmit multiple *Rickettsia* species via vertical transmission. This has been demonstrated, for instance, by the exclusion or inhibition of the transovarial transmission of *Rickettsia rickettsii* by *Rickettsia peacockii* in *Dermacentor andersoni* [44], of *Rickettsia rhipicephali* by *R. montanensis* in *Dermacentor variabilis* [45] and of *R. rickettsii* by *Rickettsia bellii* in *Amblyomma dubitatum* [46]. Krawczak et al. [38] drew a similar inference when comparing the populations of *Rickettsia parkeri* and *Candidatus Rickettsia andeanae* in *A. tigrinum* in southern Brazil.

This phenomenon, called "rickettsial interference," occurs when the initial infection by one species of Rickettsia prevents or partially inhibits ovarian infection by another species. Although the exact mechanism of this interference is still not known [45], such microbiological interaction may play a significant role in the distribution of pathogenic rickettsial species, and the diseases associated with ixodid ticks.

Thus, due to the nature of the vectors associated with *Ca. R. andeanae*, especially its interaction with A. parvum, a vector of low specificity and considerable anthropophily, new studies are needed to confirm the organism's lack of pathogenicity, as well as its role as a possible inhibitor of pathogenic species.

Rickettsia Amblyommatis

The last member of the SFGR identified in the study, *Rickettsia amblyommatis* (known as *Candidatus Rickettsia amblyommii* until 2016), is a cosmopolitan rickettsia, transmitted both transovarially and transstadially, and can be found in a wide array of ticks throughout the Americas, including several species from the genera *Amblyomma*, *Rhipicephalus*, and *Dermacentor* [47,48].

Originally considered non-pathogenic due to the absence of clinical signs after inoculation in guinea pigs, it is now understood that its pathogenicity seems to be related to the infecting strain. More recent studies, which used different strains of the species, demonstrated clinical signs such as testicular enlargement, dermatitis, moderate fever, and behavioral changes, along with findings such as hepatic necrosis and increased antibody titers, indicating an immune response [48,49].

Regarding its ability to infect humans, Richardson et al. [49], in their review, highlight reports of immune reactions such as skin rash, erythema migrans, and fever following bites from *Amblyomma americanum* - the primary vector of *R. amblyommatis* in the United States - as well as epidemiological and diagnostic findings suggesting an association between these symptoms and infection by the agent.

This scenario supports the findings of Apperson et al. [50], who detected elevated IgG titers for *R. amblyommatis* in patients presenting with a disease similar to Spotted Fever, yet without reactivity to *R. rickettsii*. Nonetheless, both Apperson et al. [50] and Richardson et al. [49] contend that these observations do not definitively establish the pathogenicity of *R. amblyommatis* in humans and emphasize the need for targeted studies to clarify this, particularly given the frequent co-occurrence of different rickettsial species in the same regions

Furthermore, as appears to be the case with *Ca. R. andeanae* and other previously mentioned species, some studies indicate that *R. amblyommatis* may reduce or inhibit the persistence of infection and transmission of other rickettsiae in vectors (rickettsial interference), particularly *R. rickettsii* and *R. parkeri*. This could potentially reduce the intensity of infection in mammals, acting as a form of protective factor, although the existing studies on this matter remain inconclusive [49,51].

In light of these considerations, the detection of *Rickettsia amblyommatis* for the first time in the state of Ceará warrants attention, particularly due to its association with three different species of ixodid ticks: *Rh. linnaei*, *A. parvum*, and *A. pseudoconcolor*. Thus, the possible pathogenic potential of *R. amblyommatis*, the significant number of vectors already associated with it, and its possible inhibitory effect on other rickettsiae [49], underscore the need for further studies. Such investigations are essential to clarify the role of this bacterium as a potential etiological agent and its relevance in maintaining the biological cycles of other rickettsial species.

Transitional Group Rickettsiae

Rickettsia felis

Originally described in 1991 and commonly associated with *Ctenocephalides felis* fleas, its primary vector and reservoir, *Rickettsia felis* is an organism with recognized pathogenicity, considered a common cause of febrile conditions in Africa, similar to murine typhus [52,53]. Due to the worldwide distribution of *C. felis*, its primary vector, and the detection of the agent in various species of ticks and mites across different parts of the globe, *R. felis* is considered a ubiquitous pathogen [54]. However, despite being confirmed as pathogenic and widely distributed, the biological characteristics and eco-epidemiology associated with the species are still not fully understood [55].

It is noteworthy that, despite its confirmed pathogenicity, broad geographic distribution, and association with various vectors, only a single case of *Rickettsia felis* has been documented in Brazil to date [56]. This is likely due to underreporting of cases, particularly considering that the clinical presentation caused by the agent is generally mild and nonspecific. Such characteristics may lead both infected patients and healthcare professionals to deem further investigation of the underlying causes unnecessary. Thus, the need for more research on the pathogen's biology and its role in public health is evident, especially when considering the worldwide distribution of its vectors.

Rickettsia asembonensis

Rickettsia asembonensis is a bacterium that belongs to the group of R. felis-like organisms (RFLOs) within the Transitional Group of Rickettsia, transmitted by ticks and fleas [57]. Originally identified in Kenya in 2013, the species has since been detected in various arthropods collected from vertebrate hosts around the world, as noted by Loyola et al. [58].

Although understanding of its pathogenicity is still in its early stages, with infections caused by the organism generally considered asymptomatic, some studies have already pointed to a correlation between the detection of the agent in humans and cases of acute fever, suggesting a pathogenic potential, even in the Amazon Basin [58,59]. Furthermore, studies by Silva et al. [60] and Bitencourth et al. [61] have already linked its presence to ticks in Brazil, and the present study further underscores the need for attention to this bacterium, having found the rickettsia in association with Rh. lin[illegible]i ticks on Ceará state for the first time, thus showing its expansion.

CONCLUSION

Rhipicephalus linnaei, Amblyomma parvum, Amblyomma longirostre, Amblyomma nodosum, Amblyomma ovale, and Amblyomma pseudoconcolor are ectoparasite species found in the studied region.

This study represents the first report of the circulation of *Rickettsia amblyommatis* and *Rickettsia asembonensis* in potential vectors in the state of Ceará.

It also marks the first documented association between *Amblyomma parvum* and *Rickettsia parkeri* in Brazil.

The presence of *Candidatus Rickettsia andeanae* and *Rickettsia amblyommatis* may be linked to a lower frequency of vectors infected with *R. rickettsii* and *R. parkeri* in the region. This interaction between organisms warrants furth[illegible]vestigation.

Although the pathogenicity of *R. amblyommatis* remains officially undetermined, studies suggest its potential pathogenicity, highlighting the need for further evaluation of its role as a possible etiological agent.

REFERENCES

1. Parola P, Paddock CD, Socolovschi C, Labruna MB, Mediannikov O, Kernif T, Abdad MY, Stenos J, Bitam I, Fournier PE, Raoult D. *Update on tick-borne*

rickettsioses around the world: a geographic approach. Clin Microbiol Rev. 2013 Oct;26(4):657-702. doi: 10.1128/CMR.00032-13. Erratum in: Clin Microbiol Rev. 2014 Jan;27(1):166. PMID: 24092850; PMCID: PMC3811236.

2. Martiniano NOM, Sato TP, Vizzoni VF, Ventura SF, Oliveira SV, Amorim M, Gazêta GS. *A new focus of spotted fever caused by Rickettsia parkeri in Brazil.* Rev Inst Med Trop Sao Paulo. 2022 Mar 11;64:e22. doi: 10.1590/S1678-9946202264022.
3. Thu~~hu~~, M.J. *et al. Diversity of spotted fever group rickettsiae and their association with host ticks in Japan*. *Sci Rep* 9, 1500 (2019). https://doi.org/10.1038/s41598-018-37836-5
4. Blanton LS. *The Rickettsioses: A Practical Update.* Infect Dis Clin North Am. 2019 Mar;33(1):213-229. doi: 10.1016/j.idc.2018.10.010. PMID: 30712763; PMCID: PMC6364315.
5. Moura-Martiniano NO, Machado-Ferreira E, Cardoso KM, Gehrke FS, Amorim M, Fogaça AC, Soares CA, Gazêta GS, Schumaker TT. *Rickettsia and vector biodiversity of spotted fever focus, Atlantic rain forest biome, Brazil.* Emerg Infect Dis. 2014 Mar;20(3):498-500. doi: 10.3201/eid2003.131013
6. Brasil. Ministério da Saúde. *Perfil Epidemiológico* da *Febre Maculosa no Brasil – 2013 a 2023.* 2024b. Boletim Epidemiológico, Vol. 55. Secretaria de Vigilância em Saúde e Ambiente
7. Oliveira S.V., Guimarães, JN.; Reckziegel, GC.; Neves, BM.; ARAÚJO-Vilges, KM. Fonseca, LX. VOIETTA, F.; Pereira, SVX.; CALDAS, EP.; Gazeta, GS.; Gurgel-Gonçalves, R. *An update on the epidemiological situation of spotted fever in Brazil*. J Venom Anim Toxins Incl Trop Dis. 2016a 22;22(1):22

8. Szabó MPJ, Pinter A, Labruna MB. *Ecology, Biology and distribution of spotted fever tick vectors in Brazil.* Front Cell Infect Microbiol 2013; 3: 1-9.
9. Pacheco RC, Moraes-Filho J, Guedes E, Silveira I, Richtzenhain LJ, Leite RC, Labruna MB. *Rickettsial infections of dogs, horses and ticks in Juiz de Fora, southeastern Brazil, and isolation of Rickettsia rickettsii from Rhipicephalus sanguineus ticks.* Med Vet Entomol. 2011 Jun;25(2):148-55. doi: 10.1111/j.1365-2915.2010.00915. x.
10. Pinter, A.; Costa, CS.; Holcman, M.M.; Camara, M., Leite, R.M. (organizadores). *A Febre Maculosa Brasileira na Região Metropolitana de São Paulo*. BEPA 2016; 13 (151): 3-47.
11. Aguirre AAR, Garcia MV, Costa IND, Csordas BG, Rodrigues VDS, Medeiros JF et al. *New records of ticks- associated spotted fever group Rickettsia in Amazon-Savannanh ecotone, Brazil.* Ticks Tick Borne Dis., 2018, 9 (4): 1038- 44
12. Moerbeck L, Vizzoni VF, Machado-ferreira E, Cavalcante RC, Oliveira SV, Soares CAG, Amorim M, Gazeta GS. *Rickettsia (Rickettsiales: Rickettsiaceae) Vector Biodiversity in High Altitude Atlantic Forest Fragments Within a Semiarid Climate: A New Endemic Area of Spotted-Fever in Brazil*. J Med Entomol. 2016.
13. Cavacalnte, RC, Pivisan NCA, Silva, LOR, Moura, FBP, Gomes, RGA, Oliveira, RP, et al. *Implantação da Vigilância Ambiental das Riquetsioses no Estado do Ceará no período de 2010 a 2022*. J. Health Biol. Sci. v12i1.4718.p1-7.2024
14. Amorim, M.; Serra-freire, NM. *Chave dicotômica para identificação de larvas de algumas espécies do gênero Amblyomma Koch, 1844 (Acari: Ixodidae).* Entomologia y Vectores 1999.

15. Martins, TF.; Onofrio, VC.; Barros-Battesti, D.; Labruna, MB. *Nymphs of the genus Amblyomma (Acari: Ixodidae) of Brazil: descripitions, redescriptions and identification key*. Ticks Tick Borne Dis. 2010.

16. Barros-Battesti, DM.; Arzua, M.; Bechara, GH. *Carrapatos de Importância Médico-Veterinária da Região Neotropical: Um guia ilustrado para identificação de espécies.* São Paulo: Vox; 2006.

17. Dantas-Torres F, Fernandes Martins T, Muñoz-Leal S, Onofrio VC, Barros-Battesti DM. *Ticks (Ixodida: Argasidae, Ixodidae) of Brazil: Updated species checklist and taxonomic keys.* Ticks Tick Borne Dis. 2019 Oct;10(6):101252. doi: 10.1016/j.ttbdis.2019.06.012. Epub 2019 Jun 17.

18. Aljanabi, SM.; Martinez, I. *Universal and rapid salt-extraction of high-quality genomic DNA for PCR-based techniques*. Nucl. Acids. Res. 1997.

19. Labruna MB, Whitwhorth T, Horta MC, Bouyer DH, Mcbride JW, Pinter A, et al. *Rickettsia species infecting Amblyomma cooperi ticks from an area in the state of São Paulo, Brazil, where Brazilian spotted fever is endemic*. J Clin Microbiol, 2004a

20. Labruna MB, Mcbride JW, Bouyer DH, Camargo LMA, Camargo EP, Walker DH. *Molecular evidence for a spotted fever group Rickettsia species in the tick Amblyomma longirostre in Brazil*. Journal of Medical Entomology, 2004b

21. Choi YJ, Lee SH, Park KH, Koh YS, Lee KH, Baik HS, Choi MS, Kim IS, Jang WJ. *Evaluation of PCR-based assay for diagnosis of spotted fever group rickettsiosis in human serum samples.* Clin Diagn Lab Immunol. 2005 Jun;12(6):759-63. doi: 10.1128/CDLI.12.6.759-763.2005.

22. Sambrook, J.; Russell, D.W. Molecular Cloning: A Laboratory Manual, 3rd ed.; Cold Spring Harbor: New York, NY, USA, 2001

23. THOMPSON J, HIGGINS D, GIBSON T. CLUSTAL W. *Improving the sensitivity of progressive multiple sequence alignment through sequence weighting, position-specific gap penalties and weight matrix choice.* Nucleic Acids Res. 1994; 22: 4673-90.

24. TAMURA K, STECHER G, PETERSON D, FILIPSKI A, KUMAR S. *MEGA 6: molecular evolutionary genetics analysis version 6.0.* Mol Biol Evol. 2013; 30: 2725-9.

25. GOUY M, GUINDON S, GASCUEL O. *SeaView version 4: a multiplatform graphical user interface for sequence alignment and phylogenetic tree building.* Mol Biol and Evol. 2010; 27 (2): 221-4.

26. GUINDON S, DUFAYARD JF, LEFORT V, ANISIMOVA M, HORDIJK W, GASCUEL O. *New algorithms and methods to estimate maximum-likelihood phylogenies: assessing the performance of PhyML 3.0.* Syst Biol. 2010; 59: 307-21.

27. Anisimova M, Gascuel O. *Approximate likelihood-ratio test for branches: A fast, accurate, and powerful alternative*. Syst Biol. 2006 Aug;55(4):539-52. doi: 10.1080/10635150600755453. PMID: 16785212.

28. Moerbeck L, Vizzoni VF, DE Oliveira SV, Cavalcante R, Coelho GCB, Duarte NFH, Amorim M, Gazêta GS. *Rickettsia sp. Strain NOD Infecting Ticks (Amblyomma nodosum) in an Endemic Area of Spotted Fever in Brazil*. J Wildl Dis. 2018

29. NIERI-BASTOS FA, MARCILI A, DE SOUSA R, PADDOCK CD, LABRUNA MB. *Phylogenetic Evidence for the Existence of Multiple Strains of Rickettsia parkeri in the New World.* Appl Environ Microbiol. 2018 Apr 2;84(8):e02872-17. doi: 10.1128/AEM.02872-17

30. SILVA-RAMOS, C. R., HIDALGO, M., & FACCINI-MARTÍNEZ, Á. A. *Clinical, epidemiological, and laboratory features of Rickettsia parkeri rickettsiosis: A systematic review.* Ticks and Tick-Borne Diseases*, 12(4), 2021 101734.* doi: 10.1016/j.ttbdis.2021.101734

31. SANTIBÁÑEZ S, PORTILLO A, SANTIBÁÑEZ P, PALOMAR AM, OTEO JA. *Usefulness of rickettsial PCR assays for the molecular diagnosis of human rickettsioses.* Enferm Infecc Microbiol Clin. 2013 May;31(5):283-8. doi: 10.1016/j.eimc.2012.08.001.

32. PIRANDA, E.M., FACCINI, J.L.H., PINTER, A., PACHECO, R.C., CANC¸ADO, P.H.D. & LABRUNA, M.B. *Experimental infection of Rhipicephalus sanguineus ticks with the bacterium Rickettsia rickettsii, using experimentally infected dogs.* Vector-borne and Zoonotic Diseases, 2010. doi:10.1089/vbz.2009.0250.

33. DANTAS-TORRES, F.; de SOUSA-PAULA, LC.; & OTRANTO, D. *The Rhipicephalus sanguineus group: updated list of species, geographical distribution, and vector competence*. Parasites Vectors 17, 540 2024. https://doi.org/10.1186/s13071-024-06572-3

34. SÁNCHEZ-MONTES S, SALCEDA-SÁNCHEZ B, BERMÚDEZ SE, AGUILAR-TIPACAMÚ G, BALLADOS-GONZÁLEZ GG, HUERTA H, AGUILAR-DOMÍNGUEZ M, MORA JD, LICONA-ENRÍQUEZ JD, MORA DD, LÓPEZ-PÉREZ AM, TORRES-CASTRO MA, ALCÁNTARA-RODRÍGUEZ V, BECKER I, COLUNGA-SALAS P. *Rhipicephalus sanguineus* Complex in the Americas: Systematic, Genetic Diversity, and Geographic Insights. Pathogens. 2021 Sep 1;10(9):1118. doi: 10.3390/pathogens10091118.

35. Guglielmone AA, ROBBINS RG. *Hard Ticks (Acari: Ixodida: Ixodidae) Paralizing Humans: A Global Overview*. London: Springer; 2018.
36. OGRZEWALSKA, M., LITERAK, I., MARTINS, T. F., & LABRUNA, M. B. *Rickettsial infections in ticks from wild birds in Paraguay.* Ticks and Tick-Borne Diseases*,* 2014. *5(2), 83–89.* doi: 10.1016/j.ttbdis.2013.08.004
37. Noden BH, Roselli MA, Loss SR. *Rickettsia parkeri and Candidatus Rickettsia andeanae in Amblyomma maculatum Group* Ticks. Emerg Infect Dis. 2020 Feb;26(2):371-374. doi: 10.3201/eid2602.190664.
38. Krawczak FS, Binder LC, Gregori F, Martins TF, Pádua GT, Sponchiado J, Melo GL, Polo G, Labruna MB. *'Candidatus Rickettsia andeanae' and Probable Exclusion of Rickettsia parkeri in Ticks from Dogs in a Natural Area of the Pampa Biome in Brazil.* Pathogens. 2023 Mar 12;12(3):446. doi: 10.3390/pathogens12030446.
39. Nieri-Bastos FA, Lopes MG, Cançado PHD, Rossa GAR, Faccini JLH, Gennari SM et al. *Candidatus Rickettsia andeanae, a spotted fever group agent infecting Amblyomma parvum ticks in two Brazilian biomes.* Mem Inst Oswaldo Cruz 2014;109(2):259–61.
40. Lugarini, C., T. F. Martins, M. Ogzewalska, N.C.T. Vasconcelos, V. A. Ellis, J. B. Oliveira, A. Pinter, M. B. Labruna, and J.C.R. Silva. *Rickettsial agentes in avian ixodid ticks in northeast Brazil.* Ticks Tick-Borne Dis. 2015. 6: 364–375
41. Ferrari FA, Goddard J, Moraru GM, Smith WE, Varela-Stokes AS. *Isolation of "Candidatus Rickettsia andeanae" (Rickettsiales: Rickettsiaceae) in embryonic cells of naturally infected Amblyomma maculatum (Ixodida: Ixodidae)*. J Med Entomol. 2013 Sep;50(5):1118-25. doi: 10.1603/me13010. PMID: 24180118.

42. Grasperge BJ, Morgan TW, Paddock CD, Peterson KE, Macaluso KR. *Feeding by Amblyomma maculatum (Acari: Ixodidae) enhances Rickettsia parkeri (Rickettsiales: Rickettsiaceae) infection in the skin*. J Med Entomol. 2014 Jul;51(4):855-63. doi: 10.1603/me13248.

43. Paddock CD, Denison AM, Dryden MW, Noden BH, Lash RR, Abdelghani SS, et al. *High prevalence of "Candidatus Rickettsia andeanae" and apparent exclusion of Rickettsia parkeri in adult Amblyomma maculatum (Acari: Ixodidae) from Kansas and Oklahoma.* Ticks Tick Borne Dis. 2015 Apr;6(3):297-302. doi: 10.1016/j.ttbdis.2015.02.001. Epub 2015 Mar 13.

44. Burgdorfer, W.; Hayes, S.F.; Mavros, A.J. Nonpathogenic rickettsiae in *Dermacentor andersoni*: A limiting factor for the distribution of *Rickettsia rickettsii*. In *Rickettsiae and Rickettsial Diseases*; Burgdorfer, W., Anacker, R.L., Eds.; Academic Press: New York, NY, USA, 1981; pp. 585–594.

45. Macaluso, K.R.; Sonenshine, D.E.; Ceraul, S.M.; Azad, A.F. *Rickettsial infection in Dermacentor variabilis (Acari: Ixodidae) inhibits transovarial transmission of a second Rickettsia*. J. Med. Entomol. 2002, *39*, 809–813.

46. Sakai, RK., Costa, FB., Ueno, TEH., Ramirez, DG., Soares, JF., Fonseca, AH., Labruna, MB., Barros-Battesti, DM. *Experimental infection with Rickettsia rickettsii in an Amblyomma dubitatum tick colony, naturally infected by Rickettsia bellii.* Ticks and Tick-Borne Diseases, 2014. *5(6), 917–923.* doi: 10.1016/j.ttbdis.2014.07.003

47. Labruna MB, Mattar S, Nava S, Bermudez S, Venzal JM, Dolz G, ET AL. *Rickettsioses in Latin America, Caribbean, Spain and Portugal*. Rev MVZ Cord 2011;16(2):2435- 57.

48. Snellgrove AN, Krapiunaya I, Scott P, Levin ML. *Assessment of the Pathogenicity of Rickettsia amblyommatis, Rickettsia bellii, and Rickettsia montanensis in a Guinea Pig Model*. Vector Borne Zoonotic Dis. 2021 Apr;21(4):232-241. doi: 10.1089/vbz.2020.2695. Epub 2021 Feb 17.

49. Richardson EA, Roe RM, Apperson CS, Ponnusamy L. *Rickettsia amblyommatis in Ticks: A Review of Distribution, Pathogenicity, and Diversity.* Microorganisms. 2023 16;11(2):493. doi: 10.3390/microorganisms11020493.

50. Apperson CS, Engber B, Nicholson WL, Mead DG, Engel J, Yabsley MJ, Dail K, Johnson J, Watson DW. *Tick-borne diseases in North Carolina: is "Rickettsia amblyommii" a possible cause of rickettsiosis reported as Rocky Mountain spotted fever?* Vector Borne Zoonotic Dis. 2008 Oct;8(5):597-606. doi: 10.1089/vbz.2007.0271.

51. Wright, CL., Sonenshine, DE., Gaff, HD., & Hynes, WL. *Rickettsia parkeri Transmission to Amblyomma americanum by Cofeeding with Amblyomma maculatum (Acari: Ixodidae) and Potential for Spillover.* Journal of Medical Entomology, 2015 *52(5), 1090–1095.* doi:10.1093/jme/tjv086

52. Brown LD, Macaluso KR. *Rickettsia felis, an Emerging Flea-Borne Rickettsiosis*. Curr Trop Med Rep. 2016; 3:27-39. doi: 10.1007/s40475-016-0070-6. Epub 2016 Apr 23.

53. Liodaki, M.; Angelakis, E.; Spanakos, G.; Papadogiannaki, I.; Samarkos, M.; Daikos, GL.; et al. *Rickettsial Agents Associated with Ectoparasites in Attica, Greece.* Zoonotic Dis. 2022, *2*, 247-257. https://doi.org/10.3390/zoonoticdis2040020

54. Tsokana CN, Kapna I, Valiakos G. *Current Data on Rickettsia felis Occurrence in Vectors, Human and Animal Hosts in Europe: A Scoping Review.*

Microorganisms. 2022 Dec 16;10(12):2491. doi: 10.3390/microorganisms10122491.

55. Mcginn J, Lamason RL. *The enigmatic biology of rickettsiae: recent advances, open questions and outlook.* Pathog Dis. 2021 Apr 9;79(4):ftab019. doi: 10.1093/femspd/ftab019.
56. Raoult D, La Scola B, Enea M, Fournier P-E, Roux V, Fenollar F, Galvão MAM, Lamballerie X. A flea associated *Rickettsia* pathogenic for humans. Emerging Infectious Diseases 7:73-81, 2001.
57. Maina AN, Jiang J, Luce-Fedrow A, ST John HK, et al. *Worldwide presence and features of flea-borne Rickettsia asembonensis.* Front Vet Sci. 2018; 5:334. doi:10.3389/fvets.2018.00334.
58. Loyola S, Palacios-Salvatierra R, Cáceres-Rey O, Richards AL. *Rickettsia asembonensis Isolated from Four Human Cases with Acute Undifferentiated Febrile Illness in Peru.* Pathogens. 2024; 13(6):489. https://doi.org/10.3390/pathogens13060489
59. Kocher C, Morrison AC, Leguia M, Loyola S, Castillo RM, Galvez HA, et al. Rickettsial disease in the Peruvian Amazon basin. PLoS Negl Trop Dis. 2016;10:e0004843. 10.1371/journal.pntd.0004843
60. Silva, AB., Vizzoni, VF., Costa, AP., Costa, F. B., Moraes-Filho, J., Labruna, MB., et al. (2017). *First report of a Rickettsia asembonensis related infecting fleas in Brazil.* Acta Tropica*, 172, 44–49.* doi: 10.1016/j.actatropica. 2017.04
61. Bitencourth K, Amorim M, de Oliveira SV, Voloch CM, Gazêta GS. *Genetic diversity, population struture and rickettsias in Amblyomma ovale in areas of epidemiological interest for spotted fever in Brazil.* Med Vet Entomol. 2019; 33(2):256-68.

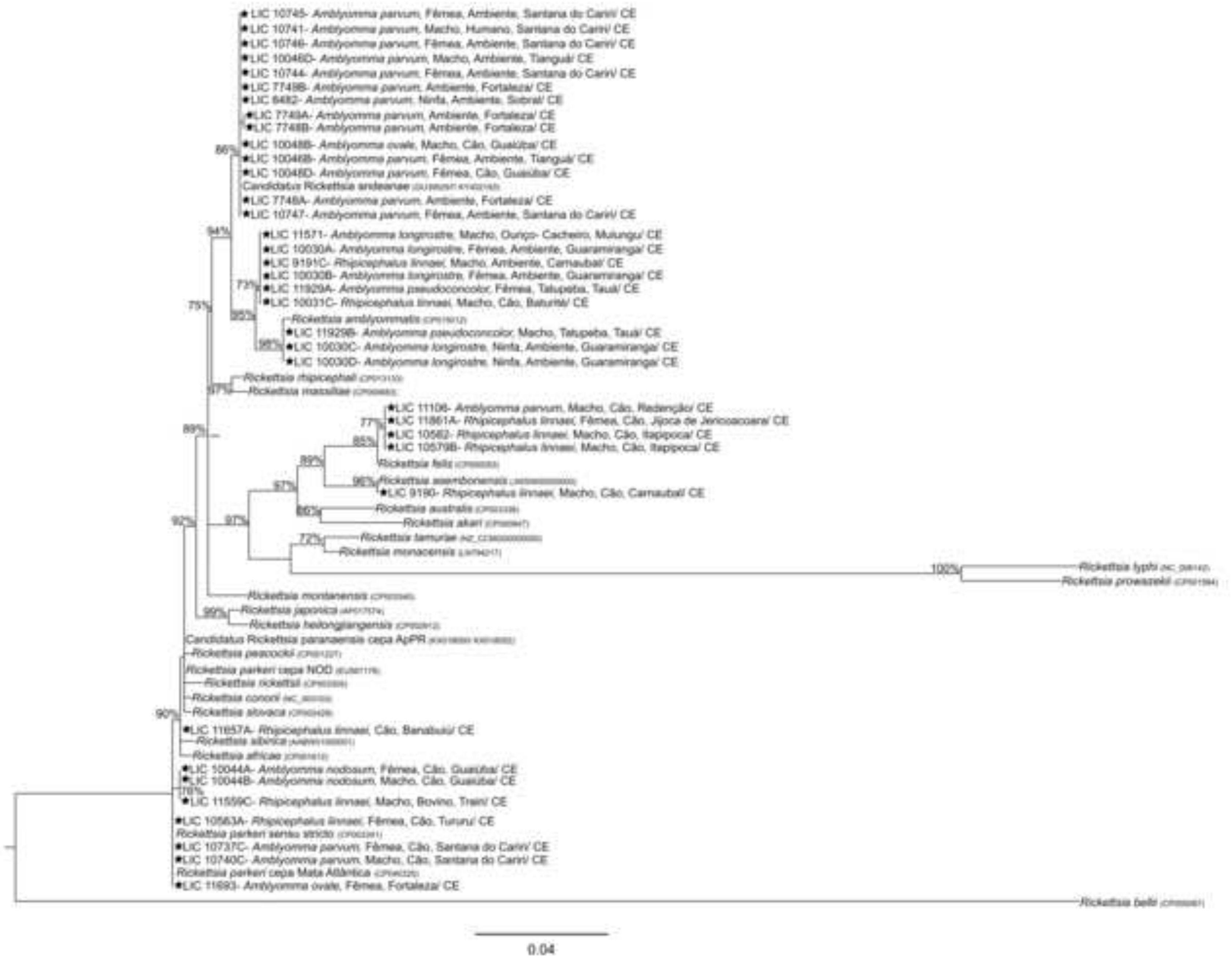
★LIC 10745- *Amblyomma parvum*, Fêmea, Ambiente, Santana do Cariri/ CE
★LIC 10741- *Amblyomma parvum*, Macho, Humano, Santana do Cariri/ CE
★LIC 10746- *Amblyomma parvum*, Fêmea, Ambiente, Santana do Cariri/ CE
★LIC 10046D- *Amblyomma parvum*, Macho, Ambiente, Tianguá/ CE
★LIC 10744- *Amblyomma parvum*, Fêmea, Ambiente, Santana do Cariri/ CE
★LIC 7749B- *Amblyomma parvum*, Ambiente, Fortaleza/ CE
★LIC 6482- *Amblyomma parvum*, Ninfa, Ambiente, Sobral/ CE
★LIC 7749A- *Amblyomma parvum*, Ambiente, Fortaleza/ CE
★LIC 7748B- *Amblyomma parvum*, Ambiente, Fortaleza/ CE
86%
★LIC 10048B- *Amblyomma ovale*, Macho, Cão, Guaiúba/ CE
★LIC 10046B- *Amblyomma parvum*, Fêmea, Ambiente, Tianguá/ CE
★LIC 10048D- *Amblyomma parvum*, Fêmea, Cão, Guaiúba/ CE
Candidatus Rickettsia andeanae
★LIC 7748A- *Amblyomma parvum*, Ambiente, Fortaleza/ CE
★LIC 10747- *Amblyomma parvum*, Fêmea, Ambiente, Santana do Cariri/ CE
94%
★LIC 11571- *Amblyomma longirostre*, Macho, Ouriço- Cacheiro, Mulungu/ CE
★LIC 10030A- *Amblyomma longirostre*, Fêmea, Ambiente, Guaramiranga/ CE
★LIC 9191C- *Rhipicephalus linnaei*, Macho, Ambiente, Carnaubal/ CE
★LIC 10030B- *Amblyomma longirostre*, Fêmea, Ambiente, Guaramiranga/ CE
73%
★LIC 11929A- *Amblyomma pseudoconcolor*, Fêmea, Tatupeba, Tauá/ CE
★LIC 10031C- *Rhipicephalus linnaei*, Macho, Cão, Baturité/ CE
75%
95%
Rickettsia amblyommatis
★LIC 11929B- *Amblyomma pseudoconcolor*, Macho, Tatupeba, Tauá/ CE
98%
★LIC 10030C- *Amblyomma longirostre*, Ninfa, Ambiente, Guaramiranga/ CE
★LIC 10030D- *Amblyomma longirostre*, Ninfa, Ambiente, Guaramiranga/ CE
Rickettsia rhipicephali
97%
Rickettsia massiliae
★LIC 11106- *Amblyomma parvum*, Macho, Cão, Redenção/ CE
77%
★LIC 11861A- *Rhipicephalus linnaei*, Fêmea, Cão, Jijoca de Jericoacoara/ CE
★LIC 10582- *Rhipicephalus linnaei*, Macho, Cão, Itapipoca/ CE
89%
85%
★LIC 10579B- *Rhipicephalus linnaei*, Macho, Cão, Itapipoca/ CE
89%
Rickettsia felis
96%
Rickettsia asembonensis
97%
★LIC 9190- *Rhipicephalus linnaei*, Macho, Cão, Carnaubal/ CE
86%
Rickettsia australis
92%
97%
Rickettsia akari
72%
Rickettsia tamurae
Rickettsia monacensis
100%
Rickettsia typhi
Rickettsia prowazekii
Rickettsia montanensis
99%
Rickettsia japonica
Rickettsia heilongjiangensis
Candidatus Rickettsia paranaensis cepa ApPR
Rickettsia peacockii
Rickettsia parkeri cepa NOD
Rickettsia rickettsii
Rickettsia conorii
Rickettsia slovaca
90%
★LIC 11657A- *Rhipicephalus linnaei*, Cão, Banabuiú/ CE
Rickettsia sibirica
Rickettsia africae
★LIC 10044A- *Amblyomma nodosum*, Fêmea, Cão, Guaiúba/ CE
★LIC 10044B- *Amblyomma nodosum*, Macho, Cão, Guaiúba/ CE
76%
★LIC 11559C- *Rhipicephalus linnaei*, Macho, Bovino, Trairi/ CE
★LIC 10563A- *Rhipicephalus linnaei*, Fêmea, Cão, Tururu/ CE
Rickettsia parkeri sensu stricto
★LIC 10737C- *Amblyomma parvum*, Fêmea, Cão, Santana do Cariri/ CE
★LIC 10740C- *Amblyomma parvum*, Macho, Cão, Santana do Cariri/ CE
Rickettsia parkeri cepa Mata Atlântica
★LIC 11693- *Amblyomma ovale*, Fêmea, Fortaleza/ CE
Rickettsia bellii
0.04
