# Supplementary material for: The mosaic of Rickettisae from the Caatinga Biome, Brazil: New records and first interactions
Source: PLoS Negl Trop Dis. 2025 Nov 10;19(11):e0013670. doi: 10.1371/journal.pntd.0013670 (PMC12614789; doi:10.1371/journal.pntd.0013670)
Supplement: Attachment — Submitted filename: Response to Reviewers.docx [file pntd.0013670.s002.docx]

**Dear Editors and Reviewers,**

We sincerely thank you for the thorough evaluation of our manuscript. We are grateful for the constructive comments and suggestions, which have helped us significantly improve the clarity and quality of our work.

Below, we provide a detailed, point-by-point response to each of the reviewers' comments. Reviewer comments are presented in bold, followed by our responses in plain text. All modifications made in the manuscript are marked in bright blue.

Additionally, we made a few changes on our own initiative. Most notably, the phylogenetic tree (Figure 1) has been revised to improve its visual clarity and scientific accuracy. We adjusted the layout for better readability, verified the labeling of both study sequences and reference strains, and ensured that support values are clearly presented. While the phylogenetic relationships remain unchanged, the revised figure improves interpretability and more effectively illustrates how the sequences obtained in this study cluster within established *Rickettsia* species groups. This enhancement strengthens the support for our taxonomic identifications and better contextualizes the findings within the broader *Rickettsia* phylogeny.

**Reviewer #1:**

**1. Please provide a map indicating where the samples were collected and where the laboratory work was conducted. The locations seem to be quite far apart, so additional clarification is required to confirm the validity of identification process.**

We have included a map of Ceará State (Figure 2) showing the municipalities where tick samples were collected and the *Rickettsia* species identified during the study. We confirm that taxonomic identification and molecular analyses were carried out at LAC- Fiocruz, which is located in the state of Rio de Janeiro. This arrangement follows the structure of the National Environmental Surveillance System for Rickettsioses and Other Tick-Borne Diseases (RNVADTC), which designates reference laboratories such as LAC to receive and process samples from various states. All samples sent to LAC were accompanied by standardized collection forms containing detailed metadata (host, location, date, and collection method), ensuring traceability and consistency. If the reviewer still deems it necessary, we will provide a map or a description of the distance between states in the manuscript.

**2. Are the same researchers involved in both the analysis and the sample collection? How can you verify that the methods used were flannel dragging and direct picking?**

Thank you for this important observation. We clarify that some members of the research team were directly involved in the field collection of tick samples. Furthermore, all personnel involved in sample collection—whether affiliated with this study or acting under the local health authorities—received prior training from LAC–FIOCRUZ staff on standardized procedures for ectoparasite capture. These trainings are conducted in accordance with the official protocol of the National Environmental Surveillance System for Rickettsioses and Other Tick-Borne Diseases (RNVADTC), which mandates the use of validated techniques such as flannel dragging and direct picking from hosts.

Moreover, detailed data provided by the State Health Secretariat of Ceará (SESA/CE), including collection method, host, location, and date, accompanied each sample, in line with the RNVADTC protocol. This ensures the traceability and integrity of the collection process. If the editorial board deems it necessary, this information can be explicitly included in the 'Collection and Screening of Ectoparasite Samples' section."

**3. How were the tick samples preserved after collection?**

All tick samples were stored in analytical grade isopropanol (≥99% purity) immediately after collection, following the standard procedure recommended by RNVADTC for long-term DNA preservation and taxonomic work. This information was added to the "Collection and Screening of Ectoparasite Samples" section

**4. When was the taxonomic identification performed? Was the identification conducted after the sample collection between 2017 and 2023, or was it done recently after the samples were retrieved from the repository?**

Taxonomic identification was performed at the time of each sample’s arrival at the LAC–FIOCRUZ laboratory, following each collection event between 2017 and 2023. The identification was conducted using appropriate taxonomic keys for each developmental stage and was recorded in the sample metadata upon processing. If the editorial board deems it necessary, this information can be explicitly included in the 'Collection and Screening of Ectoparasite Samples' section."

**5. How were the samples stored, and at what temperature?**

After taxonomic identification and cataloging, samples were stored in analytical grade isopropanol (≥99% purity) at -20°C in the LAC–FIOCRUZ repository until DNA extraction and further analyses. This information was added to the end of the "Collection and Screening of Ectoparasite Samples" section.

**6. How the samples were transported from the repository to the lab?**

If the reviewer is referring to transport from the field collection sites to the laboratory, samples were fixed in isopropyl alcohol (>99% purity) immediately after collection and transported in UN3373-compliant containers for biological samples, in accordance with biosafety protocols. If the question refers instead to transport from the repository to the lab, we clarify that the repository is housed within the laboratory itself, and no external transport was required in that context.

**7. Some of the paragraphs lack clarity. Please rewrite it to clarify the details especially the new PCRs method. Detailed comments are provided in the attachment.**

We agree and appreciate this comment and have revised the paragraphs related to PCR methods for clarity and flow. Specifically, the rationale for using nested primers and the circumstances surrounding DNA degradation were more clearly articulated to avoid confusion. The paragraph has been rewritten, and we hope this revised version makes our methodological approach clearer and address any the confusion noted in the original phrasing.

**8. The results presented focus on the distribution and analysis of molecular detection of Rickettsia spp. infections in vectors. Information about the hosts was also included; however, there was no additional information regarding environmental factors. If you do not plan to discuss the variations and differences related to biomes, microregions, and mesoregions, please remove this sentence. Detailed comments can be found in the attachment.**

We appreciate this valuable comment and have revised the sentence accordingly. The response to the issue raised is two-fold:

**Regarding Micro and Mesoregions**: The terms "microregions" and "mesoregions" are officially used by the Brazilian Institute of Geography and Statistics (IBGE) to refer to administrative divisions within the Brazilian states. We included these terms in the manuscript to enhance the precision of location/geographical references, enabling both Brazilian and international researchers to identify specific areas more accurately. We believe this is particularly important as the ecoepidemiological knowledge of *Rickettsia* and Spotted Fever in northeastern Brazil, the Caatinga biome and Ceará state is still limited and so we propose correcting the manuscript to make it clear that the micro and mesoregions mentioned are refer strictly to administrative divisions. However, if these terms are deemed unnecessary or confusing, we are willing to remove the sentence.

**Regarding Biomes**: While we did include some host-related information in the manuscript, we acknowledge the absence of a detailed discussion on environmental factors.

**The overall discussion and conclusion was satisfactory, focusing on the disease distributions found in this study and their comparisons to other studies. However, this study solely focused on detecting Rickettsia infections in archived tick samples. A revised title should accurately represent the scope of the study.**

While we acknowledge that the study is based on archived tick samples and does not encompass the entire Caatinga biome, we believe that maintaining this geographical reference in the title remains appropriate and meaningful. The vast majority of Ceará State — where all samples were collected — lies within the Caatinga, and the collection sites represent a significant portion of this biome’s ecological range. Furthermore, research on *Rickettsia* spp. and their tick vectors in the Caatinga remains scarce and geographically uneven. By explicitly framing the study within this biome, we aim to contribute to the limited but growing body of knowledge about rickettsial ecology in semiarid northeastern Brazil and to promote greater visibility of this underrepresented region in international discussions on tick-borne diseases.

To address the reviewer’s concern regarding the geographic scope of sampling, we have revised the title to use “from the Caatinga Biome” instead of “in” or “within.” This wording more accurately reflects that our findings are based on samples collected from a portion of the biome (specifically Ceará State) without implying complete biome-wide coverage, while still maintaining ecological and regional relevance.

**This manuscript requires major revisions. The overall analyses conducted are adequate for publication; however, the authors need to restructure the entire manuscript from the title to the conclusion. The title somewhat overclaims the data that are not presented in the manuscript.**

**The authors presented their findings on the prevalence of Rickettsia spp. infections from tick samples obtained from various hosts. This information could be valuable to local stakeholders. However, the presentation of the results lacks clarity. Out of a total of 3,098 ticks collected, only 1,359 were processed, and all positive samples were sourced from localities in Ceará. Additionally, there was no analysis regarding environmental factors, and the interaction between Rickettsia parkeri and Amblyomma parvum was only briefly mentioned in one paragraph. The authors should consider revising the title for better clarity. Detailed comments can be found in the attachment.**

We thank the reviewer for the constructive feedback and for recognizing the potential value of our findings to local stakeholders. In response to the concerns raised, we have made several key revisions to improve the clarity and scope of the manuscript:

**Clarification of Sample Processing:** We have clarified in the Materials and Methods section the rationale for processing a subset (1,362) of the total 3,098 collected ticks. This subset was selected based on preservation quality and taxonomic representation, particularly focusing on genera with known epidemiological relevance for *Rickettsia* spp.

**Geographic Scope:** We have emphasized in both the Introduction and Discussion that the positive samples reported were indeed from localities within Ceará.

**Environmental Factors:** We acknowledge the reviewer’s valuable suggestion regarding environmental factors. However, we respectfully note that this aspect lies beyond the primary scope and objectives of the present study, which focused on molecular detection and species identification of *Rickettsia* spp. in archived tick samples. While we recognize the ecological importance of such factors, a meaningful integration of environmental variables would require additional data and analytical space that exceeds the current manuscript’s framework. As such, we agree that this is an important avenue for future research and intend to explore these environmental aspects more deeply in future studies.

**Rickettsia parkeri and Amblyomma parvum Interaction:** We appreciate the suggestion to elaborate on this novel finding. However, due to limited sample availability, we were unable to investigate this interaction further and have therefore reported it as an observation rather than drawing broader conclusions. In light of this, we have revised the title to avoid overstating the significance or implications of the *R. parkeri–A. parvum* association.

**Title Revision:** We acknowledge the reviewer's concern regarding the current title and its alignment with the study’s content. However, we respectfully maintain that referencing the Caatinga biome is appropriate given that Ceará’s territory falls wholly within this biome, and our findings contribute to the limited but growing body of research from this region. To address the concern about geographic scope while retaining the ecological framing, we have revised the title to:

**“The Mosaic of Rickettsiae from the Caatinga Biome, Brazil: New Records and First Interactions.”**

This formulation avoids overextending the study’s spatial claims and does not overemphasize the novel detection of *R. parkeri* in *A. parvum*, while still highlighting the broader ecological relevance of the study. That said, we would be glad to consider minor adjustments to the title for greater clarity, should the editorial team or reviewers deem it appropriate.

**Reviewer #2:**

**The study objective is poorly formulated.**

**The results presented in the study are unclear and require restructuring for ease of reading.**

We acknowledge the reviewer’s concern and have revised the Results section to enhance clarity and organization. Host information was added to Table 1, the layout of Figure 1 was restructured to improve visualization, and minor adjustments to the language were made to improve overall readability.

**Fig. 1 is unclear. Please tell us what new information this figure adds to the study.**

The comment on Fig. 1 is valid, and we clarify its purpose as follows: Fig. 1 presents the phylogenetic relationships among *Rickettsia* sequences detected in this study and reference strains, constructed using maximum likelihood methods. The tree provides essential information regarding the genetic identity and classification of the *Rickettsia* spp. detected, demonstrating how they cluster within different *Rickettsia* groups and species. This visualization supports the molecular findings and situates our sequences within the broader phylogenetic context of the genus *Rickettsia*. To enhance clarity, we have revised the figure layout, improved labeling, and expanded the legend to facilitate interpretation.

**The manuscript “The Mosaic of Rickettisae in Caatinga Biome, Brazil: New Interactions between Rickettsia parkeri and Amblyomma Parvum” is an interesting research work and an original idea, which provides information on Rickettsia species from ticks in Caatinga, Brazil. The manuscript requires extensive revision.**

We thank the reviewer for their positive assessment of our manuscript and for recognizing its originality and relevance in providing new information on *Rickettsia* species in the Caatinga biome. We also acknowledge the reviewer’s recommendation for extensive revisions. Accordingly, we have carefully revised the manuscript to enhance its clarity, structure, and methodological explanations. All reviewer comments were addressed point by point, and the necessary adjustments have been made throughout the text. We hope these changes significantly improve the quality and readability of our work.

**Reviewer #3:**

**The use of PCR assays is appropriate but the rationale for how and why they were used is not clearly explained. The section that starts: “The shift in primers used was due to their characteristics…” is confusing, because the second set of primers used for sequencing are the nested ones, which would be more sensitive, based on the description, than the assays used for screening. The statement “Thus, new PCRs for both and new positive samples were conducted using nested primers, chosen for their higher detection sensitivity…” At least with respect to the use of ompB, it differentiates between similar SFG species better than the gltA or htrA protocols, which I would think would be its primary benefit in using it for the sequencing reactions here.**

We thank the reviewer for highlighting the need to clarify the rationale behind our PCR assay choices. We have revised the relevant paragraph to explain that while *gltA* and *ompA* PCRs were used for initial screening, sequencing of these fragments was often impaired due to degradation of older samples. To standardize the molecular workflow and enhance detection sensitivity, we employed nested PCRs targeting *htrA* and *ompB* for species confirmation—hence the shift in primers. Additionally, *ompB* was selected for its superior ability to discriminate between closely related spotted fever group species. We hope this revised explanation more accurately reflects our methodological approach and resolves the confusion noted in the original phrasing.

**1.Table 1 and Table 2 are redundant—Table 1 is just the row tabulations of Table I believe Table 1 can be eliminated.**

We understand the reviewer’s observation and concern regarding potential redundancy. However, we believe that Table 1 serves a distinct purpose by presenting the number and species of hosts from which potential Spotted Fever (SF) vectors were collected. In contrast, Table 2 details the distribution and taxonomic identification of the ectoparasites collected from these hosts, without indicating the number of individual hosts involved. We consider this host-level information relevant, particularly because dogs—although frequently parasitized—are not typically emphasized in the Brazilian context as key reservoirs or indicators for Rickettsia spp. infections by veterinary or medical professionals. Therefore, we respectfully suggest retaining Table 1 to provide a more complete epidemiological perspective, with host common names inserted as recommended.

**2. Recommend using common host names along with scientific for host names in the tables.**

We agree and have inserted the hosts common names on table 1 alongside scientific names in the tables to improve clarity and accessibility for a broader audience.

**3.Table 3: Headings in Portuguese**

The reviewer is correct, and we have addressed this by translating all column headings in Table 3 into English to ensure consistency and clarity throughout the manuscript. We appreciate the attention to detail.

**4.In the statement “Of these 37 sequenced samples, 24 were analyzed for the ompB gene, from a total of 25 amplified by Nested PCR, and 21 were analyzed for the htrA gene, from a total of 23 amplified by Nested PCR.”: I am confused about what this means. Does this mean that 25 of the ompB samples produced bands and 24 were sequenced successfully, and that 23 of the htrA produced bands, of which 21 were sequenced successfully, or something else? Please clarify. It would also be helpful to indicate the number of samples that were sequenced for both ompB and htrA.**

We thank the reviewer for this important observation. To clarify, 25 samples produced positive bands for *ompB* by nested PCR, and 24 of these were successfully sequenced. Likewise, 23 samples amplified for *htrA*, of which 21 yielded high-quality sequencing results. Among these, 8 samples were successfully sequenced for both *ompB* and *htrA* fragments. This clarification has been added to the revised manuscript to ensure transparency and improve comprehension of our sequencing dataset, and the section of the paragraph rewritten accordingly. The alterations made can be seen in the Results section.

**5. “Notably, of the 37 sequences obtained…”: This should be “samples that were successfully sequenced”, not number of sequences, correct?**

The reviewer is correct, and we appreciate the observation. We have revised the sentence to state “samples that were successfully sequenced” instead of “sequences obtained,” to more accurately reflect the data and avoid potential misinterpretation.
