# Supplementary material for: The mosaic of Rickettisae from the Caatinga Biome, Brazil: New records and first interactions
Source: PLoS Negl Trop Dis. 2025 Nov 10;19(11):e0013670. doi: 10.1371/journal.pntd.0013670 (PMC12614789; doi:10.1371/journal.pntd.0013670)
Supplement: Attachment — Submitted filename: Review comments_PLOSNTD.docx [file pntd.0013670.s004.docx]

**Review comments – PLOS NTD**

The authors of this manuscript discuss an important topic on rickettsiosis and ticks. It adds new findings to the tick population and their potential associated rickettsial pathogens. Few things need to be attended to before the manuscript is accepted.

1. **Introduction**

Adequate and relevant.

2. **Methodology**

- Need more clarity and details
- Needs to be re-written with clear subheadings on setting, sampling procedures (collection, storage, transport etc) , research design, sample processing (clearly differentiating morphological identification, physical processing of ticks, primary molecular detection, DNA sequencing etc)
- There is no mention of sequence submission to GeneBank and acquisition of accession number?
- There is no mention of anything on ethics and approvals, or any sort of waivers if available. This needs to be mentioned.

3. **Results**

- Elaborate and adequate
- Figure of Phylogenetic tree need a better and more clear picture

4. **Discussion**

- Adequate

5. **Overall comments**

The authors need to run through and read the manuscript in view of checking all spellings, use of capital letters for words in between sentences since a few of these were observed. The manuscript also needs to be formatted as per the author guideline. Even some track changes are still visible.
