# Supplementary material for: The mosaic of Rickettisae from the Caatinga Biome, Brazil: New records and first interactions
Source: PLoS Negl Trop Dis. 2025 Nov 10;19(11):e0013670. doi: 10.1371/journal.pntd.0013670 (PMC12614789; doi:10.1371/journal.pntd.0013670)
Supplement: Attachment — Submitted filename: Response to Reviewers 2.docx [file pntd.0013670.s005.docx]

**Dear Editors and Reviewers,**

We sincerely thank you for the thorough evaluation of our manuscript. We are grateful for the constructive comments and suggestions, which have helped us significantly improve the clarity and quality of our work.

Below, we provide a detailed, point-by-point response to each of the reviewers' comments. Reviewer comments are presented in bold, followed by our responses in plain text. All modifications made in the manuscript are marked in bright blue.

**Reviewer #1:**

**The method has undergone significant improvement, now following a clear and easy-to-understand flow.**

R: We thank the reviewer for this positive assessment and are glad that the revisions to the Methods section improved its clarity and organization.

**The results are well presented, organized, and easier to understand. I just need a few more clarifications as below:**

**How is the confirmation of the host species conducted?
Are ectoparasites identified solely through morphological identification?**

R: We appreciate the reviewer’s positive assessment and are glad to provide clarification.

Host species were confirmed by field teams during collection, based on direct observation of the captured vertebrates, and the information was documented in the standardized metadata forms provided by the Ceará State Health Secretariat (SESA/CE) to LAC-Fiocruz.

Ectoparasite identification was indeed carried out using morphological criteria only, but this was performed under stereomicroscopy and following multiple established and widely used taxonomic keys (References 14-17): Amorim and Serra-Freire (for larvae), Martins et al. (for nymphs), and Barros-Battesti et al. and Dantas-Torres et al. (for adults). These references provide robust diagnostic features, and all taxa identified in this study correspond to species that are common and well documented in Brazil. Given these conditions, morphological identification was considered sufficient for the purposes of this work.

**Why did u suggest this conclusion? "The presence of Candidatus Rickettsia andeanae and Rickettsia amblyommatis may be linked to a lower frequency of vectors infected with R. rickettsii and R. parkeri in the region". I recommend not including this sentence in the conclusion, as the study focuses solely on reporting the prevalence of the pathogens.**

R: We understand the reviewer’s concern regarding the scope of our conclusions. However, we respectfully believe that retaining this statement in a revised form is appropriate, as our study discusses interspecific interactions among *Rickettsia* species throughout the text. The intent was not to present a definitive claim, but rather to provide a contextual interpretation that highlights potential ecological dynamics which could help explain regional differences. Specifically, the presence of *Ca. R. andeanae* and *R. amblyommatis* in the Northeast may help contextualize why *R. rickettsii* and *R. parkeri* appear less frequently compared to the Southeast of Brazil, where they are well recognized as agents of endemic disease. To ensure clarity, we have rephrased this sentence to explicitly present it as a hypothesis arising from our findings and the broader literature, rather than as a firm conclusion, thereby avoiding overstating its implications while still acknowledging its ecological relevance. We hope the revised phrasing satisfactorily addresses the reviewer’s concern

**Reviewer #4:**

**The overall methods have been improved and are clear. One potential important missing element is information on IACUC or any veterinary health regulatory board approval. This may not be required in this instance, but I know some organizations are more stringent than others. If a veterinary health board approval was obtained, please add it the first part of the methods section.**

R: We appreciate the reviewer’s attention to this important issue. In this case, specific IACUC or veterinary health board approval was not required. All samples analyzed in this study were obtained from the LAC repository, where they had been archived following official surveillance campaigns. These ectoparasite specimens were originally collected during environmental surveillance activities and/or suspected spotted fever case investigations coordinated by the State Health Secretariat of Ceará (SESA/CE), under the framework of Brazil’s National Environmental Surveillance System for Rickettsioses and Other Tick-Borne Diseases (RNVADTC). Within this framework, LAC–FIOCRUZ serves both as a research laboratory and as part of the national diagnostic network for arthropod-borne diseases. Therefore, the use of repository samples and associated data for research purposes falls within the scope of government surveillance activities, and no additional ethical approval was required. If the reviewer considers it helpful, we are willing to add a clarifying statement to the *Methods* section.

**This section (results) has been much improved, and no further recommendations are noted.**

R: We are grateful for the reviewer’s positive feedback and are pleased that the revisions to the Results section have improved its clarity and presentation.

**Two important notes/comments are missing from the discussion section. The authors need to specifically discuss the limitation that they had poor genetic material in older ticks (as mentioned in the results section) and that this could lead to an underreporting or missed cases. Additionally, the authors should discuss the lack of R. rickettsii in their ticks. I was surprised they didn't find this pathologically important species, and a brief discussion should be noted (pontificate why this species may not be in the local environment).**

R: We appreciate the reviewer’s thoughtful comments and will address both separately:

**1.** With regard to the limitations of the genetic material, we would like to respectfully note that this issue has already been addressed in the *Methods* section (originally under *Detection and Characterization of Bioagents*, now revised as *DNA Extraction, Amplification and Sequencing*). There, we explained the challenges associated with degraded genetic material in older samples and the rationale for applying nested PCRs (*htrA* and *ompB*) to standardize detection across all specimens. As the primary objective of the study was to characterize the diversity of *Rickettsia* species, rather than to investigate the causes of DNA degradation or storage-related factors, we believe that expanding this discussion further would fall outside the intended scope of the manuscript. e hope that the revisions already incorporated help to clarify our methodological approach and that the focus of the manuscript remains clear and relevant to its objectives. That said, we are of course open to further guidance from the reviewer if additional clarification is deemed necessary.

Regarding the concern of potential underreporting, we note that all samples included in this study were originally processed for diagnostic purposes upon arrival at the laboratory, and results were reported at that time to the Brazilian Ministry of Health. The present study represents a retrospective molecular re-analysis of archived material, and therefore, while sequencing success was not achieved for all previously positive samples, this does not alter the original diagnostic reporting. Importantly, this study’s focus is not on prevalence estimates, but on documenting the range of *Rickettsia* species detected. For these reasons, we respectfully suggest that additional discussion on this technical limitation would not add substantially to the manuscript’s objectives.

**2**. We appreciate the reviewer’s interest in this point. We chose not to expand on the absence of *R. rickettsii* in detail because this finding is consistent with the current epidemiological knowledge of the region itself. The main vector of *R. rickettsii* in Brazil is *Amblyomma sculptum*, a species that is not considered endemic to the Northeast region, including Ceará state. While sporadic reports of *R. rickettsii* have been made in certain municipalities of the region, these remain exceptions. Spotted fever caused by *R. rickettsii* is primarily an endemic issue in southeastern and parts of southern Brazil, and its occurrence in the Northeast appears to be rare and focal. Moreover, a recent systematic review and meta-analysis (Ribeiro et al., 2021) found that only approximately 2% of ticks in Brazil were reported as infected with *R. rickettsii*, highlighting that its detection is uncommon even at the national scale. We therefore believe that the absence of this species in our dataset is consistent with the broader epidemiological context and does not require further discussion within the scope of this manuscript. For these reasons, we considered the absence of *R. rickettsii* in our samples expected, and thus did not highlight it in the Discussion. If the editors consider it important, we are willing to add a brief statement clarifying this ecological context.

**Reviewer #5:**

**Need more clarity and details - Needs to be re-written with clear subheadings on setting, sampling procedures (collection, storage, transport etc), research design, sample processing (clearly differentiating morphological identification, physical processing of ticks, primary molecular detection, DNA sequencing etc)**

R: We acknowledge the reviewer’s suggestion and have thoroughly revised the *Methods* section to improve clarity and structure. The section has now been reorganized with clear subheadings that reflect the workflow of the study, covering: Vector Collection and Transport, Morphological Identification and Storage, DNA Extraction, Amplification and Sequencing, and Molecular Data Analysis. This restructuring separates fieldwork, morphological identification, laboratory processing, and molecular analysis into distinct sections, making the methodology more transparent and easier to follow. We believe these changes address the reviewer’s concern and improve the overall readability of the manuscript. **- There is no mention of sequence submission to GeneBank and acquisition of accession number? There is no mention of anything on ethics and approvals, or any sort of waivers if available. This needs to be mentioned.**

R: We thank the reviewer for raising this important point. We confirm that all required information was included in the submission package and clarify it here for transparency:

1. **Sequence submission:** The genetic data supporting the findings of this study have already been submitted to the NCBI GenBank database. The sequences will be made publicly available upon acceptance/publication of the manuscript under the accession numbers **PV085523–PV085565**.
2. **Competing interests and financial disclosures:** All authors have completed and submitted the required disclosure forms to the journal at the time of resubmission.
3. **Ethics approval/waiver:** Specific IACUC or veterinary health board approval was not required, as no new collections or animal handling were performed by the authors, and all samples analyzed in this study were obtained from the LAC repository, where they had been archived following official surveillance activities. The ectoparasite specimens had been collected in previous years during official environmental surveillance campaigns and/or suspected spotted fever case investigations coordinated by the State Health Secretariat of Ceará (SESA/CE), within the framework of Brazil’s National Environmental Surveillance System for Rickettsioses and Other Tick-Borne Diseases (RNVADTC). Within this framework, LAC–FIOCRUZ functions both as a research laboratory and as part of the national diagnostic network for arthropod-borne diseases. Thus, the use of repository samples and associated data for research purposes falls under government surveillance activities, and no additional ethical approval was required. If the editors consider it necessary, we are willing to add a clarifying statement to the *Methods* section.

**-Elaborate and adequate; - Figure of Phylogenetic tree need a better and more clear picture (Results)**

R: We are grateful for the reviewer’s positive assessment and are pleased that the Results section was found appropriate. In response to the comment, we have revised the phylogenetic tree (Figure 1) to improve its clarity and resolution within the journal parameters, and we hope these adjustments provide a clearer and more satisfactory visualization.
